# Risk factors of in-stent restenosis among coronary artery disease patients with syphilis undergoing percutaneous coronary intervention: a retrospective study

**DOI:** 10.1186/s12872-021-02245-6

**Published:** 2021-09-15

**Authors:** Ling Zhang, Yu Wang, Zhe Zhang, Hongyuan Liang, Liang Wu, Liang Ni, Guiju Gao, Di Yang, Hongxin Zhao, Jiang Xiao

**Affiliations:** 1 Clinical and Research Center of Infectious Diseases, Beijing Ditan Hospital, Capital Medical University, Beijing, China; 2 Beijing Fuxing Hospital, Capital Medical University, XiCheng District, Beijing, China

**Keywords:** Syphilis, Percutaneous coronary intervention, Coronary artery disease, In-stent restenosis, Population attributable risk

## Abstract

**Background:**

The risk factors of in-stent restenosis (ISR) among coronary artery disease (CAD) patients with syphilis after percutaneous coronary intervention (PCI) are not fully understood. Therefore, this study aimed to elucidate not only the risk factors of ISR among CAD patients with syphilis after performing PCI, but also the population attributable risk percentage (PAR%), which is used to quantify the proportion of ISR that could be eliminated if particular risk factors are not present.

**Methods:**

Evaluation of the prevalence, risk factors, and their PAR% for ISR among CAD patients with syphilis undergoing PCI was conducted retrospectively at Beijing Ditan Hospital. CAD patients with syphilis underwent PCI from August 2010 to August 2019 and received a diagnosis, coronary angiography, PCI, and periodical follow-up. The clinical, laboratory, and imaging data were reviewed and summarised anonymously from electronic medical records. The chi-square or Fisher exact test was used in data analysis.

**Results:**

Among 114 CAD patients with syphilis undergoing PCI, ISR occurred in 18 patients (15.78%). The multivariate Cox regression model indicated that average stent length ≥ 35 mm (adjusted hazard ratio [HR] = 4.47, 95% confidence interval [CI] = 1.30–15.44, *p* = 0.018) and titres of the toluidine red unheated serum test (TRUST) > 1:16 (adjusted HR = 3.72, 95% CI = 1.22–11.36, *p* = 0.021) were associated with an increased risk of ISR, while successful antisyphilitic treatment (adjusted HR = 0.12, 95% CI = 0.02–0.95, *p* = 0.045) was protective predictor of ISR among these patients. The PAR% values of particular risk factors associated with ISR including average stent length ≥ 35 mm, titres of TRUST > 1:16, and successful antisyphilitic treatment were 12.2%, 24.0%, and -39.6%, respectively, among these patients.

**Conclusions:**

Preventing the occurrence of ISR among CAD patients with syphilis undergoing PCI requires clinical intervention. Our results indicated that carefully evaluating the length of the vessel lesion to determine whether the stent length is < 35 mm, prioritising the clinical intervention for titres of TRUST > 1:16, and providing successful antisyphilitic treatment could reduce the risk of ISR occurrence.

## Background

Coronary intervention or percutaneous coronary intervention (PCI) is an important therapeutic method for coronary artery disease (CAD) [1], and in-stent restenosis (ISR) is the most common complication of PCI, which occurs in 3%–20% of patients undergoing coronary stent implantation [2]. ISR is a key risk factor that affects the prognosis of PCI and is a difficult problem associated with coronary intervention of CAD [3].

Syphilis is caused by infection with the pathogen *Treponema pallidum subsp. pallidum*, which is a curable sexually transmitted disease. As economic development and migrants moved to China in recent decades, syphilis re-emerged gradually and widely spread among men who have sex with men, female sex workers, drug users, and HIV-infected populations, so it has become an important public health problem in China [4]. Despite its curability, some individuals are unaware of their infection due to latent syphilis until cardiovascular diseases occur and confirmed by further laboratory examination of *Treponema pallidum* particle agglutination (TPPA) and titres of the toluidine red unheated serum test (TRUST) [4]. PCI is emergently conducted among CAD patients with syphilis, and ISR may occur in these patients, although antisyphilitic treatment is recommended after performing PCI.

Previous studies focused on finding the risk factors of ISR among general CAD patients receiving PCI [5, 6], but risk factors of ISR among CAD patients with syphilis after PCI are not fully understood. This study aims to elucidate not only risk factors of ISR among CAD patients with syphilis but also the population attributable risk (PAR) percentage (PAR%), which is used to quantify the proportion of ISR that could be eliminated if particular risk factors are not present and reminds physicians to prioritise interventions based on these risk factors to reduce ISR development among CAD patients with syphilis.

## Methods

### Ethical considerations

This observational study was conducted at Beijing Ditan Hospital, Capital Medical University, the largest national referral hospital of sexually transmitted infections and infectious diseases, and the clinical ethics review committee of the hospital approved the retrospective study procedure of using anonymised laboratory, imaging, and clinical data. The need for informed consent was waived because of the retrospective nature of the study.

### Study design and population

To elucidate not only the risk factors of ISR among CAD patients with syphilis after performing PCI, but also the PAR%, we included CAD patients with syphilis who underwent PCI from August 2010 to August 2019 and received a diagnosis, coronary angiography, PCI, and periodical follow-up at Beijing Ditan Hospital in this retrospective study. The clinical, laboratory and imaging data were reviewed and summarised anonymously from electronic medical records. The patients undergoing stent implantation received periodical follow-up, and the presence of ISR was evaluated based on angiography at 12 months after PCI or at any time the patient had clinical symptoms such as chest pain. The risk factors and their PAR% for ISR among patients with syphilis undergoing PCI were evaluated based on these clinical data.

### Percutaneous coronary angiography, postoperative treatment, and in-stent restenosis

Two experienced interventional cardiologists performed coronary angiography based on expert consensus on PCI [7], and appropriately selected generation 2 stents, but not generation 1 or bare metal stents according to the angiography results. Before PCI, the patients were required to receive 300 mg of oral aspirin and 300 mg of clopidogrel. During surgery, an infusion of low-molecular-weight heparin (70–100 IU/kg) was administered for anticoagulation, and after surgery, low-molecular-weight heparin (4000 IU) twice daily was continuously administered for 3–5 days, along with clopidogrel (75 mg per day) for 12 months and aspirin (100 mg per day) indefinitely. Additionally, it was recommended that patients receiving PCI take some medications, including angiotensin-converting enzyme inhibitors and statins. In this study, the CAD patients with syphilis were also required to receive standard antisyphilitic treatment after PCI.

Coronary angiography follow-up was conducted 12 months after PCI or when clinical symptoms and signs indicated the presence of ischaemia. ISR was defined when the diameter of stenosis was ≥ 50% inside of the stent at the follow-up angiography or stenosis was 5-mm proximal or distal to the stent, based on recommendation of expert consensus published by the European Society of Cardiology [7].

### Data collection

Demographic data included age and sex, and clinical data included smoking history, alcoholic use, documented risk factors of CAD (hyperlipidaemia, hypertension, diabetes, and chronic kidney diseases [CKD]), and medical information of baseline PCI coronary angiography (target vessel lesion sites and numbers, implanted stent styles, and average stent length and diameter). Laboratory data included a lipid panel (including triglyceride [TG], total cholesterol[TC], low-density lipoprotein[LDL], high-density lipoprotein [HDL]), homocysteine [HCY]), TPPA, and titres of TRUST at baseline PCI and ISR occurrence. Clinical treatment data included the intervention for risk factors of CAD (including lipid-lowering, antihypertensive, and glucose-lowering agents), dual antiplatelet therapy (DAPT), and antisyphilitic treatment with benzathine penicillin after undergoing PCI.

### Clinical definitions

A successful antisyphilitic treatment had a serological follow-up at 3, 6, 12, 18 and 24 months after therapy with benzathine penicillin, with titres of TRUST gradually decreasing and becoming negative [8]. Effective DAPT was defined as taking a potent P2Y12 inhibitor, clopidogrel (75 mg per day), for 12 months and aspirin (100 mg per day) indefinitely after performing PCI [7]. Multivessel disease was defined as a stenosis diameter > 50% that occurred in more than two vessels in the coronary artery system [9]. Hyperlipidaemia was defined as elevated cholesterol and/or TG levels in plasma, and included a decreased HDL cholesterol level [10]. CAD patients with syphilis in this observational cohort were referred to as coronary atherosclerotic heart disease patients with baseline serum positive TPPA and TRUST results, and some patients were combined with cardiovascular syphilis based on coronary ostial stenosis aortic valvular insufficiency according to coronary artery angiography results [11], besides atherosclerotic plaque. CKD was defined as a decline in kidney function as evidenced by a glomerular filtration rate < 60 mL/min/1.73 m^2^, or markers of kidney damage, or both for at least 3 months, regardless of the underlying cause [12].

PAR was used to describe the risk of ISR among CAD patients with syphilis undergoing PCI that can be attributed to exposure of a specific risk factor. The PAR% was used to quantify the proportion of ISR that could be eliminated if particular risk factors were not present or modified [13]. PAR% was calculated based on the following formula:

$${\text{PAR }} = {\text{ It }}{-}{\text{ Io}}$$

$${\text{PAR}}\% \, = \, \left( {{\text{It }}{-}{\text{ Io}}} \right) \, /{\text{ It }} \times { 1}00\%$$

where It is the proportion of ISR among all CAD patients with syphilis undergoing PCI and Io is the proportion of ISR among CAD patients with syphilis undergoing PCI who were not exposed to specific risk factors.

### Statistical analysis

Categorical variables are presented as percentages and were evaluated with the chi-square test or Fisher exact test. In CAD patients with syphilis, univariate Cox regression models were first used to determine the association of the following variables with the presence of ISR: age, sex, smoking history, alcoholic drinking, cardiovascular syphilis, hypertension, diabetes, dyslipidaemia, homocysteine level ≥ 15 µmol/L, titres of TRUST > 1:16, number of target vessels ≥ 3, number of implanted stents > 3, average stent length ≥ 35 mm, average stent diameter ≤ 3 mm, effective DAPT, and successful antisyphilitic treatment. Statistically significant predictors were included in the subsequent multivariate Cox regression model. According to Bruzzi et al.’s recommendation [14], the PAR% of particular risk factors associated with ISR was calculated among CAD patients with syphilis based on the Cox proportional hazard models. Additionally, a Kaplan–Meier plot was computed, and the log-rank test was conducted to determine differences in the cumulative hazard between the ISR group and non-ISR group. The clinical data were analysed statistically with SPSS 21.0 (IBM Corp., Armonk, NY, USA). A *p*-value < 0.05 was considered statistically significant in this study.

## Results

### Baseline clinical features

In this observational cohort, the baseline clinical features are presented in detail in Table 1. One hundred fourteen patients with CAD and syphilis presented with acute chest pain, and coronary angiography and PCI were urgently performed. Among those, 23 patients (20.2%) were diagnosed as having acute coronary syndrome and 91 patients (79.8%) as having chronic coronary syndrome. Additionally, serum screening of baseline TPPA and TRUST was positive prior to stent implantation, and six patients were found to be complicated with cardiovascular syphilis diagnosed based on coronary artery angiography findings. The prevalence of titres > 1:16 was significantly higher in the ISR group than that in non-ISR group (33.3% versus 8.33%, *p* = 0.010). No significant difference was found in age > 50 years, sex, cigarette smoking, hypertension, diabetes, CKD, cardiovascular syphilis, hyperlipidaemia, and HCY level, except for alcoholic consumption, between the ISR and non-ISR groups.

**Table 1:** Baseline demographics, clinical and laboratory features of ISR among CAD patients with syphilis undergoing PCI

Variables	Total n(%)	ISR n(%)	Non-ISR n(%)	*p*
Patients number	114 (100)	18 (100)	96 (100)	-
Demographic and clinical data				
Age ≥ 50 years	93 (81.6)	16 (88.9)	77 (80.2)	0.589
Gender-Male	91 (79.8)	13 (72.2)	78 (81.3)	0.578
Smoking history	77 (67.5)	13 (72.2)	64 (66.7)	0.644
Alcoholic drinking	50 (43.9)	12 (67.7)	38 (39.6)	0.034
Cardiovascular syphilis	6 (5.26)	1 (5.56)	5 (5.21)	1.000
Hypertension	65 (57.0)	9 (50.0)	56 (58.3)	0.512
Diabetes	42 (36.8)	8 (44.4)	34 (35.4)	0.466
Chronic kidney disease	2 (1.75)	0 (0.00)	2 (2.08)	1.000
Coronary angiography				
Number of target vessels				
One	30 (26.3)	2 (11.1)	28 (29.2)	0.063
Two	26 (22.8)	3 (16.7)	23 (24.0)	0.711
Multivessel disease	58 (50.9)	13 (72.2)	45 (46.9)	0.048
Target vessels				
LM	18 (15.8)	2 (11.1)	16 (16.7)	0.810
LAD	103 (90.4)	18 (100.0)	85 (88.5)	0.282
LCX	68 (59.6)	13 (72.2)	55 (57.3)	0.236
RCA	77 (67.5)	16 (88.9)	61 (63.5)	0.035
Implanted stent numbers > 3	21 (18.4)	8 (44.4)	13 (13.5)	0.006
Average stent length ≥ 35 mm	13 (11.4)	4 (22.2)	9 (9.38)	0.242
Average stent diameter ≤ 3 mm	93 (81.6)	17 (94.4)	76 (79.2)	0.229
Laboratory data				
Dyslipidemia	76 (66.7)	14 (77.8)	62 (64.6)	0.276
TG > 2.3 mmol/L	19 (16.7)	4 (22.2)	15 (15.6)	0.730
TC > 6.2 mmol/L	2 (1.75)	0 (0.0)	2 (1.75)	1.000
HDL < 1 mmol/L	64 (56.1)	12 (66.7)	52 (54.2)	0.327
LDL > 3.12 mmol/L	10 (8.77)	1 (5.56)	9 (9.38)	0.943
HCY > 15umol/L	47 (41.2)	11 (61.1)	36 (37.5)	0.062
TPPA	114 (100)	18 (100)	96 (100)	-
Titres of TRUST > 1:16	14 (12.3)	6 (33.3)	8 (8.33)	0.010
Clinical treatment				
Statins	74 (64.9)	14 (77.8)	60 (62.5)	0.213
β-Blocker	46 (40.3)	12 (66.7)	34 (35.4)	0.013
Glucose-lowering agents*	26 (22.8)	1 (5.56)	25 (26.0)	0.111
Effective DAPT	78 (68.4)	13 (72.2)	65 (67.7)	0.705
Successful antisyphilitic treatment	49 (43.0)	1 (5.56)	48 (50.0)	0.001

*Insulin was excluded

LM: left main; LAD: left anterior descending; LCX: left circumflex artery; RCA: right coronary artery; TG: triglyceride; TC: total cholesterol; HDL: high density lipoprotein; LDL: low density lipoprotein; HCY: homocysteine;TPPA: Treponema Pallidum Particle Agglutination; TRUST: Toluidine Red Unheated Serum Test; ACEI: angiotensin converting enzyme inhibitor; ARB: angiotensin receptor blocker; DAPT: dual anti-platelet therapy

In our study, 36 patients received DAPT for < 12 months due to gastrointestinal haemorrhage, while another 78 patients received DAPT for up to 12 months. Coronary angiography follow-up was conducted 12 months after PCI, and DAPT continued among patients with poor results of coronary angiography; thus, the longest duration of DAPT was up to 30 months. In addition, there was no difference in effective DAPT between the groups.

### Baseline angiographic features

The baseline angiographic features are shown in detail in Table 1. All patients received generation 2 drug-eluting stents to reduce the occurrence of early thrombosis. Patients in the ISR group had more multivessel diseases in the coronary artery system than patients in non-ISR group (72.2% versus 46.9%, *p* = 0.048). The prevalence of > 3 implanted stents was significantly higher in the ISR group than in the non-ISR group (44.4% versus 13.5%, *p* = 0.006). Patients in the ISR group had more stenosis in the right coronary artery than patients in the non-ISR group (88.9% versus 63.5%, *p* = 0.035). The other angiographic features were not significantly difference between the two groups.

### Syphilis, antisyphilitic treatment, and follow-up

The clinical features of syphilis and antisyphilitic treatment are presented in detail in Table 1. In this observational cohort, baseline TPPA and TRUST results were positive among CAD patients undergoing PCI. Although antisyphilitic treatment was recommended and periodical follow-up for the screening titres of TRUST was required, only 49 patients (43%) received successful antisyphilitic treatment. The other 65 patients did not receive successful antisyphilitic treatment, and their titres of TRUST never turned negative at follow-up.

### Prevalence of in-stent restenosis among patients with syphilis undergoing percutaneous coronary intervention

**Fig. 1:**
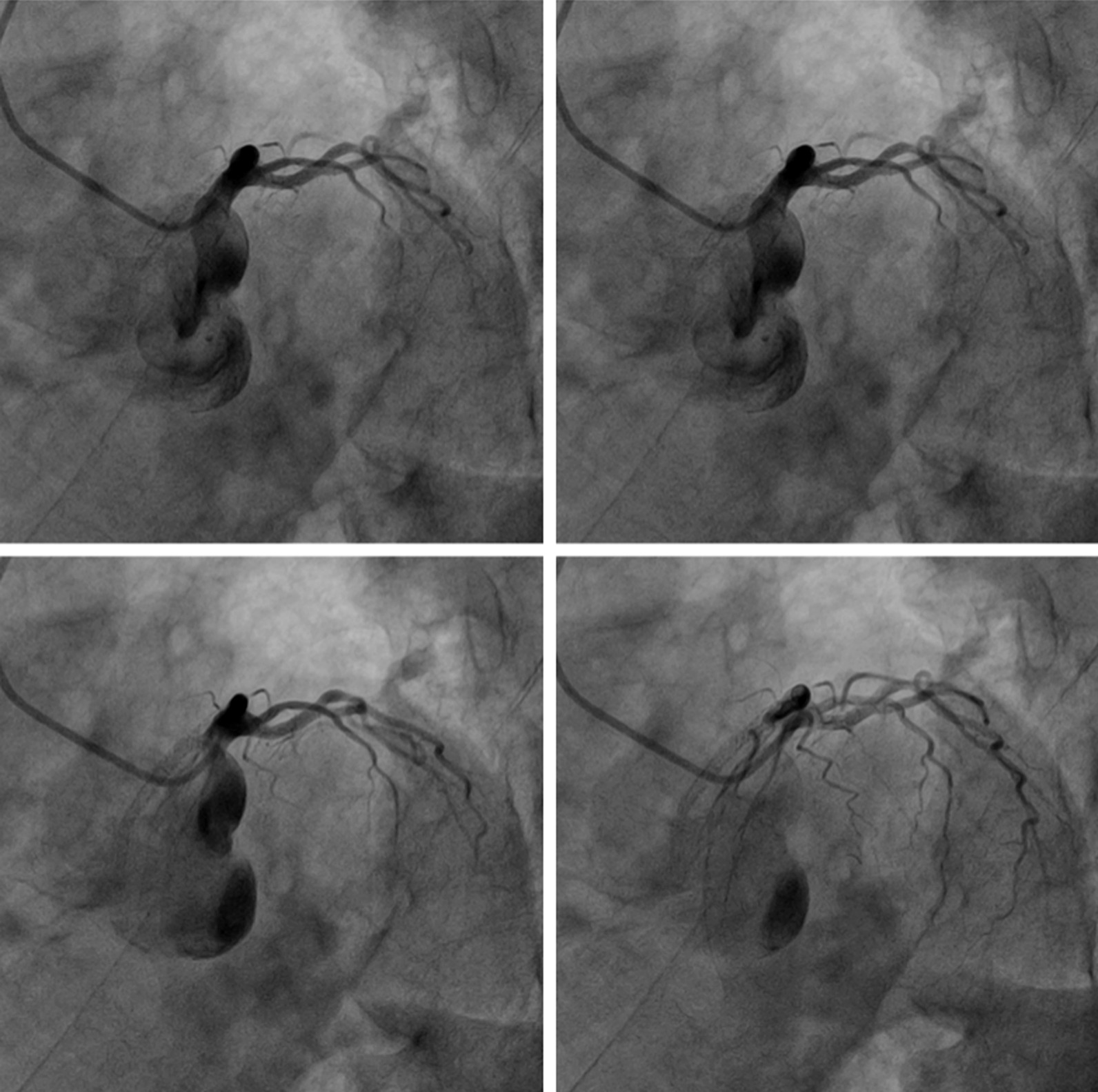
The in-stent restenosis in left anterior descending artery (LAD)

One hundred fourteen CAD patients with syphilis underwent PCI from August 2010 to August 2019 and received periodical follow-up at Beijing Ditan Hospital. ISR was occurred in 18 patients based on coronary angiography results, and the prevalence of ISR was 15.78% among CAD patients with syphilis. During the study period, 2348 patients underwent PCI at our institution, and of those, 114 patients (4.86%) were diagnosed as having syphilis. Figure 1 showed the in-stent restenosis in left anterior descending artery (LAD).

### Risk factors of in-stent restenosis among patients with syphilis undergoing percutaneous coronary intervention

As seen in Table 2, the univariate Cox regression model indicated that titres of TRUST > 1:16 (hazard ratio [HR] = 4.58, 95% confidence interval [CI] = 1.68–12.47, *p* = 0.003), > 3 implanted stents (HR = 3.31, 95% CI = 1.27–8.66, *p* = 0.015), and average stent length ≥ 35 mm (HR = 3.49, 95% CI = 1.13–10.75, *p* = 0.029) were associated with an increased odds of ISR. Additionally, successful antisyphilitic treatment (HR = 0.08, 95% CI = 0.01–0.61, *p* = 0.015) was a protective predictor of ISR among patients with syphilis undergoing PCI.

**Table 2:** Risk factors of ISR by cox proportional hazard model among study patients undergoing PCI

Variables	Unadjusted		Adjusted	
	HR (95%CI)	*p*	HR (95%CI)	*p*
Age ≥ 50 years	0.88 (0.31–2.47)	0.803	9.09 (0.87–94.63)	0.065
Gender-Male	1.94 (0.44–8.51)	0.378	0.50 (0.04–6.27)	0.594
Smoking history	1.08 (0.38–3.04)	0.888	0.96 (0.06–16.78)	0.979
Alcoholic drinking	2.65 (0.99–7.10)	0.053	4.82 (0.65–35.52)	0.123
Cardiovascular syphilis	1.07 (0.14–8.09)	0.948	4.81 (0.39–59.47)	0.221
Hypertension	0.75 (0.29–1.90)	0.541	0.68 (0.18–2.64)	0.576
Diabetes	1.32 (0.52–3.34)	0.562	1.11 (0.36–3.46)	0.860
Dyslipidemia	2.10 (0.68–6.47)	0.198	1.40 (0.35–5.67)	0.638
HCY ≥ 15umol/L	1.80 (0.70–4.67)	0.227	1.39 (0.40–4.81)	0.606
Titres of TRUST > 1:16	4.58 (1.68–12.47)	0.003	3.72 (1.22–11.36)	0.021
Number of target vessels ≥ 3	2.69 (0.95–7.60)	0.062	2.16 (0.58–8.01)	0.249
Implanted stent numbers > 3	3.31 (1.27–8.66)	0.015	1.92 (0.47–7.86)	0.364
Average stent length ≥ 35 mm	3.49 (1.13–10.75)	0.029	4.47 (1.30–15.44)	0.018
Average stent diameter ≤ 3 mm	3.26 (0.43–24.61)	0.252	1.84 (0.20–17.37)	0.595
Effective DAPT	1.08 (0.39–3.04)	0.881	1.32 (0.34–5.15)	0.694
Successful antisyphilitic treatment	0.08 (0.01–0.61)	0.015	0.12 (0.02–0.95)	0.045

ISR: in-stent restenosis; CAD:coronary artery disease; PCI:percutaneous coronary intervention; HR: Hazard Ratio; HCY:homocysteine; DAPT:dual anti-platelet therapy

The multivariate Cox regression model indicated that average stent length ≥ 35 mm (adjusted HR = 4.47, 95% CI = 1.30–15.44, *p* = 0.018) and titres of TRUST > 1:16 (adjusted HR = 3.72, 95% CI = 1.22–11.36, *p* = 0.021) were associated with an increased hazard of ISR. In addition, successful antisyphilitic treatment (adjusted HR = 0.12, 95% CI = 0.02–0.95, *p* = 0.045) was a protective predictor of ISR among patients with syphilis undergoing PCI. ISR was not predicted by age ≥ 50 years, exposure to cigarette smoking and alcoholic drinking, cardiovascular syphilis, hypertension, diabetes, hyperlipidaemia, HCY level > 15 µmol/L, number of target vessels ≥ 3, average stent diameter ≤ 3 mm, and effective DAPT.

**Fig. 2:**
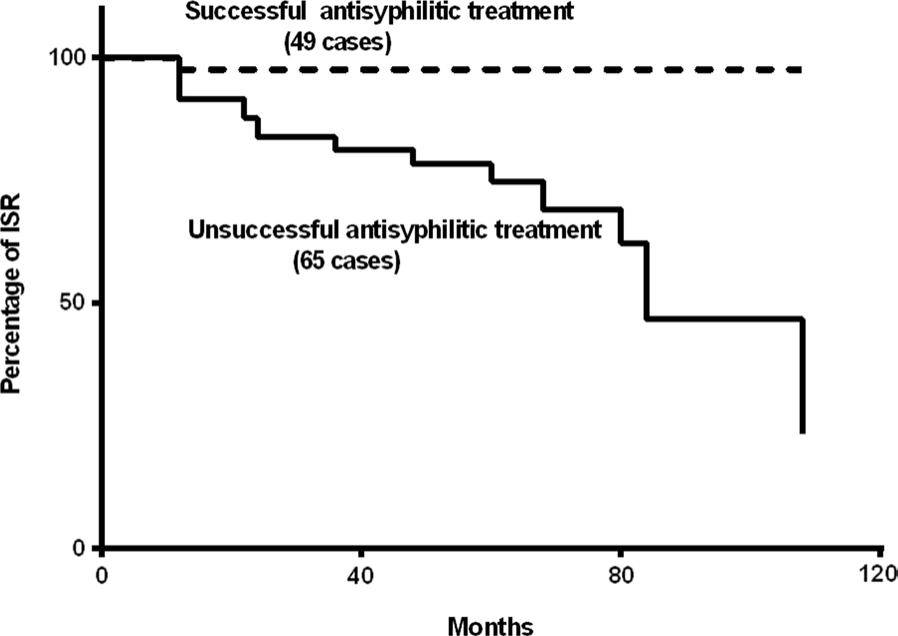
Kaplan–Meier survival curves for study patients stratified by successful or unsuccessful antisyphilitic treatment

**Fig. 3:**
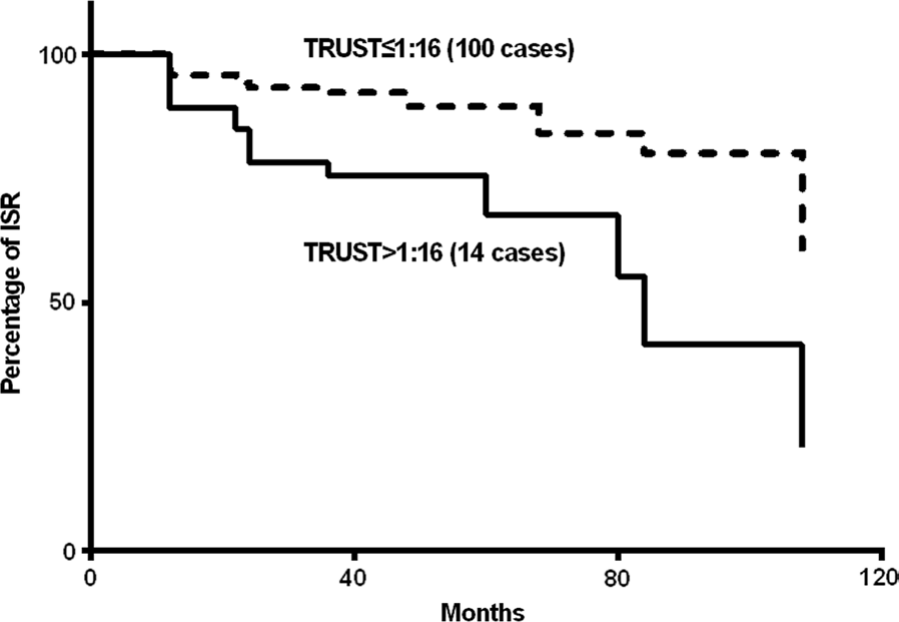
Kaplan–Meier survival curves for study patients stratified by the titres of toluidine red unheated serum test

The occurrence of clinical systems, such as chest pain, and further diagnosis of ISR by coronary angiography were observational terminal points, and the median time of follow-up was 38 months (minimum 6 months, maximum 108 months). To clarify the effects of successful antisyphilitic treatment and titres of TRUST on ISR, Kaplan–Meier survival curves were plotted for study patients stratified by the use of variables, successful/unsuccessful antisyphilitic treatment, and titres of TRUST > 1:16 or ≤ 1:16 (Figs. 2 and 3), and the log-rank test indicated that there was a significant difference between the two groups (*p* < 0.05).

### Population attributable risk percentage of risk factors of in-stent restenosis among patients with syphilis undergoing percutaneous coronary angiography

**Fig. 4:**
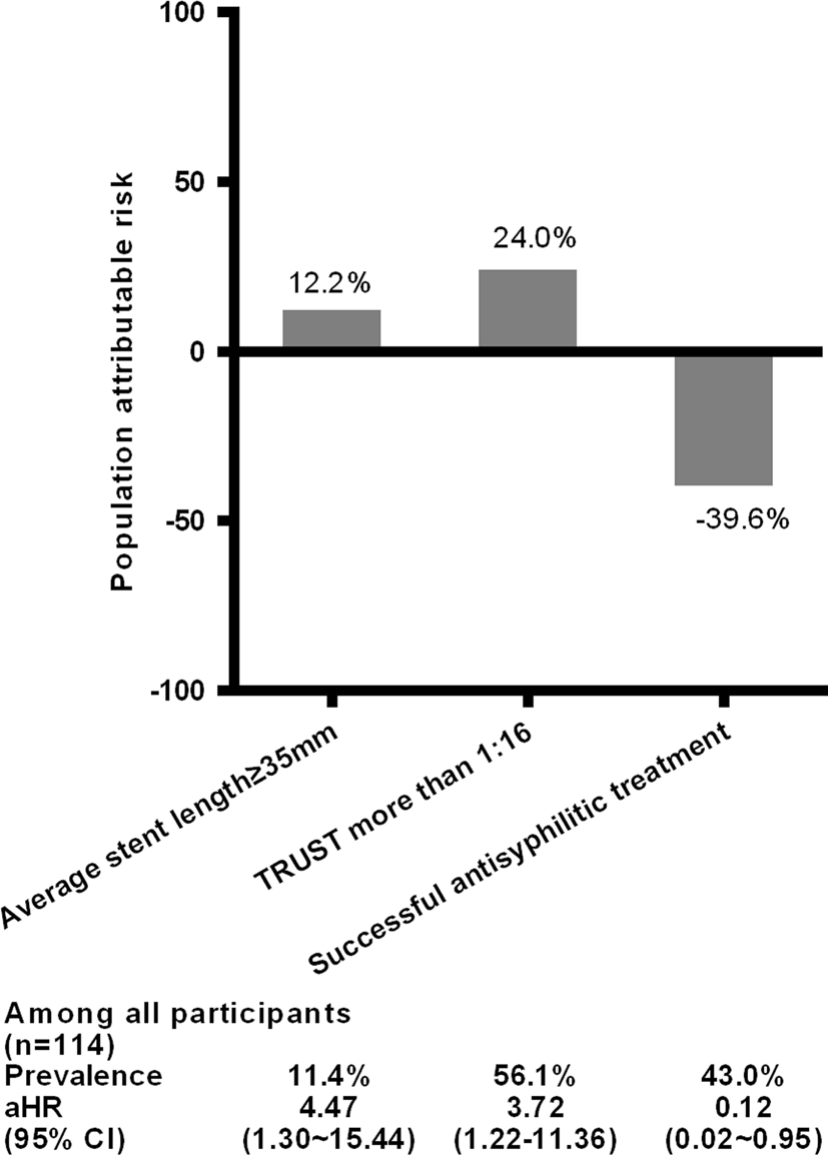
PAR% of particular risk factors associated with ISR among CAD patients with syphilis. Based on Cox proportional hazard models, the PAR% values of particular risk factors associated with ISR including average stent length ≥ 35 mm, titres of TRUST > 1:16, and successful antisyphilitic treatment are 12.1%, 24.0%, and -39.6%, respectively, among CAD patients with syphilis. Summing the PAR% of a single risk factor to derive the total PAR% of ISR among CAD patients with syphilis undergoing PCI was not recommended because there was a confounder between the risk factors in this study. PAR%, population attributable risk percentage; ISR, in-stent restenosis; CAD, coronary artery disease; TRUST, toluidine red unheated serum test; PCI, percutaneous coronary intervention

According to Bruzzi et al.’s recommendation [12], the PAR% values of particular risk factors associated with ISR include the average stent length ≥ 35 mm, titres of TRUST > 1:16, and successful antisyphilitic treatment were 12.2%, 24.0%, and -39.6%, respectively, among CAD patients with syphilis based on Cox proportional hazard models (Fig. 4).

## Discussion

ISR is a problematic complication in the treatment of CADs with PCI, as it occurred in 3%–20% of patients following stent implantation [2], and the pathogenesis of ISR has not been fully elucidated. In CAD patients with syphilis, ISR remains a difficult problem in those receiving PCI, and the prevalence of ISR among CAD patients with syphilis receiving PCI is obscure. It was reported that syphilis can affect almost any tissue or organ, including the cardiovascular system, and invasive properties of *T. pallidum* induced a delayed-type hypersensitivity response [4], resulting in syphilitic damage of the cardiovascular system, if antisyphilitic therapy was unsuccessful. In this study, we found that ISR occurred in 18 patients, with a prevalence of 15.78%. The high prevalence indicated the importance of identifying risk factors of ISR and providing earlier clinical intervention among CAD patients with syphilis who will be undergoing PCI.

In CAD patients undergoing PCI, stent thrombosis is a rare complication that can cause in-stent stenosis [14]. Cutlip et al. [15] reported that stent thrombosis occurred in < 1.0% of patients undergoing PCI and receiving routine DAPT, and Ong et al. [16] reported that 1.2% of patients with bare metal stents, 1.0% with sirolimus-eluting stents, and 1.0% with paclitaxel-eluting stents developed angiographically proven stent thrombosis in the first 30 days after stent implantation. Early stent thrombosis was attributable to mechanical problems, and although drug-eluting stents reduced the occurrence of early thrombosis by introducing local drug delivery, they increased the occurrence of late stent thrombosis due to chronic inflammatory responses, remodeling of the coronary artery vessel wall, and the progression of atherosclerosis [17–19]. In this observational cohort, generation 2 stents, not generation 1 or bare metal stents, were selected to reduce early thrombosis, and DAPT was recommended to these patients to reduce the development of late stent thrombosis based on expert consensus on PCI [7], which helped reduce the occurrence of ISR.

Among CAD patients with syphilis, if antisyphilitic therapy was overlooked, the pathogen *T. pallidum* invaded the vessel wall, including the coronary artery system, and the inflammatory response resulted in necrosis of the elastic fibers and connective tissue in vessel media and the formation of gumma, which was reactive and granulomatous [20], resulting in syphilitic damage to some degree in the coronary artery system besides atherosclerosis in these patients. In the present observational cohort, 114 CAD patients emergently received PCI and were diagnosed as having coronary atherosclerotic heart disease based on coronary artery angiography results, and the serum screening results of TPPA and TRUST were positive prior to surgery; among those, six patients were found to be complicated with syphilitic cardiovascular diseases. Although antisyphilitic treatment after performing PCI was recommended [21], most patients in this cohort obtained no or unsuccessful antisyphilitic treatment. We found that successful antisyphilitic treatment after PCI was an independent protective factor, which indicated that among CAD patients with syphilis undergoing PCI, successful antisyphilitic treatment is essential among these patients to reduce the occurrence of ISR.

ISR occurred among CAD patients undergoing PCI due to multiple risk factors, including biological, technical, mechanical, and therapeutic factors [17–19]. The risk factors of ISR among CAD patients with syphilis undergoing PCI are not fully understood. In the present study, we found that average stent length ≥ 35 mm and titres of TRUST > 1:16 were associated with an increased hazard of ISR, while successful antisyphilitic treatment was a protective predictor of ISR among CAD patients with syphilis.

The PAR% measures the burden of exposure factors and helps estimate the level of preventable diseases [22]. It assumes complete causality between a given risk factor and the pathology, so by removing the risk factor, the event probability decreases to the baseline risk. Benichou et al. [22, 23] demonstrated that the PAR% exposure factor was a causal one rather than merely related to diseases. It was reported that a longer stent length is the cause of ISR [5] and that higher titres of TRUST and unsuccessful antisyphilitic treatment [24] result in extensive vascular damage and stent thrombosis after PCI. In the present study, stent length ≥ 35 mm, titres of TRUST > 1:16, and unsuccessful antisyphilitic treatment were causal factors of ISR, which indicated that these three factors were prone to evaluation of the PAR% of ISR among CAD patients with syphilis undergoing PCI.

Chatterjee et al. [25] indicated that risk predictive models could be derived from cohort studies, in which the exposure-specific absolute risks of the disease could be estimated directly. Hu et al. [26] also reported that in a retrospective study, determining the population attributable fraction helped demonstrate the importance of promoting physical activity, a healthy diet, and other healthy lifestyles for reducing obesity among high school students in the United States, which indicated that the PAR% could be used to evaluate the burden of exposure factors in a retrospective study.

In the present study, the PAR% method was used to quantify the proportion of ISR that could be eliminated if particular hazards contributing to ISR based on Cox proportional hazard models were not present or modified, which indicated prioritisation of the clinical intervention or modification to reduce the occurrence of ISR [27]. In the current study, we calculated the PAR% values for the three aforementioned predictors to help determine the appropriate clinical intervention based on the quantitative PAR% to reduce occurrence of ISR. We also strongly recommend not summing the PAR% of a single risk factor to derive the total PAR% of ISR among CAD patients with syphilis undergoing PCI because there was a confounder between these risk factors and conditions under which this method was used [28].

Stent length is a risk factor of ISR development after PCI. Cassese et al. [29] indicated that each 10-mm increase of the stent length was a risk factor of ISR development. Cheng et al. [5] demonstrated that the longer stent resulted in more severe injury of the vessels, a stronger inflammatory reaction, and greater intimal thickness. Herein, we also found that average stent length ≥ 35 mm was an independent risk factor of ISR among CAD patients with syphilis undergoing PCI, which is similar to the findings of the aforementioned studies. We further found that the PAR% was 12.2%, which indicated that 12.2% of the risk of ISR would be reduced if average stent length ≥ 35 mm was not present or modified. This is a reminder to interventional cardiologists that among CAD patients with syphilis undergoing PCI, it is necessary to carefully evaluated the length of the vessel lesion to determine the stent length that can accurately cover the vessel lesion.

In the current study, the screening of TPPA and titres of TRUST was conducted prior to performing emergent PCI among patients with CAD, and antisyphilitic treatment was recommended after PCI. Despite the availability of effective antisyphilitic treatment, syphilis-related clinical diseases including syphilitic cardiovascular diseases and even cerebral infarction continue to cause comorbidities in Chinese syphilis-infected patients. Some patients were unaware of their syphilis infection until the occurrence of end-organ diseases, such as myocardial or cerebral infarction [30]; some patients neglected to receive antisyphilitic treatment because of mild clinical symptoms or signs; and some patients abandoned treatment because of poor psychosocial factors. In our study, despite the recommended antisyphilitic treatment and periodical follow-up for screening titres of TRUST after PCI, only 43% of patients received successful antisyphilitic treatment; the other 65 patients did not obtain successful antisyphilitic treatment, and their titres of TRUST never became negative at follow-up. The higher titres of TRUST were more prone to cause greater damage to vessels in the coronary artery system [24] and result in ISR, and we found that titres of TRUST > 1:16 was another independent risk factor of ISR among CAD patients with syphilis undergoing PCI. We further found that the PAR% was 24.0%, which indicated that 24.0% of the risk of ISR would be reduced if titres of TRUST were decreased by effective antisyphilitic treatment. We further demonstrated that successful antisyphilitic treatment after PCI was an independent protective factor for ISR, and the PAR% of successful antisyphilitic treatment was -39.6%. The aforementioned results serve to remind interventional cardiologists to attach importance to damage of the coronary artery caused by syphilis and antisyphilitic treatment in CAD patients with syphilis undergoing PCI.

Several variables, including the stent type, HCY level, and histories of diabetes, hypertension, and hyperlipidaemia, have been reported as risk factors of ISR in the general population undergoing PCI [5, 31], but we could not conclude that these variables were associated with ISR in patients with syphilis undergoing PCI. It was widely reported that the bare metal stent was associated with ISR, and experienced interventional cardiologists often selected generation 2 drug-eluting stent to avoid ISR at our hospital. Besides DAPT, clinical interventions for risk factors, including diabetes, hypertension, and hyperlipidaemia, are important and should be recommended to patients with these comorbidities. We did not find that these comorbidities were associated with ISR due to effective clinical intervention, which indicated the importance of clinical intervention for the aforementioned comorbidities in CAD patients with syphilis undergoing PCI. Although we did not find that the HCY level > 15 µmol/L and average stent diameter ≤ 3 mm were associated with ISR in our cohort, we still needed to monitor these two risk factors to alert of the occurrence of ISR.

The present study has some strengths. First, this observational study was firstly conducted to evaluate the risk factors and PAR% values of risk factors of ISR among CAD patients with syphilis undergoing PCI in China, and it is the first to quantify the proportion of ISR that could be eliminated if particular risk factors were not present or modified. Our findings will help remind physicians to prioritise interventions based on the PAR% of risk factors of ISR. Second, the most predominant finding was that successful antisyphilitic treatment was a protective factor of ISR, indicating that interventional cardiologists should attach importance to successful antisyphilitic treatment besides DAPT, which helped reduce the occurrence of ISR among CAD patients with syphilis undergoing PCI.

Our study also have some limitations. First, this study was conducted in a single centre with a small sample size, and its conclusion should be further validated at multiple centres using a prospective cohort study design. Second, potential inherent biases exist in a retrospective and observational study. Third, the duration of syphilis that affected the severity and prognosis of CAD is unknown, because we did not know the exact duration of syphilis and could not investigate the impact of syphilis duration on the occurrence of ISR.

## Conclusions

In summary, preventing the occurrence of ISR among CAD patients with syphilis undergoing PCI requires clinical intervention, and our study is the first to present the risk factors and their PAR% values of ISR among CAD patients with syphilis after PCI. We found that average stent length ≥ 35 mm and titres of TRUST > 1:16 are risk factors of ISR, and successful antisyphilitic treatment was a protective predictor of ISR. Therefore, stent length < 35 mm, prioritising clinical intervention for titres of TRUST > 1:16, and successful antisyphilitic treatment could reduce the risk of ISR occurrence by 12.2%, 24.0%, and 39.6%, respectively.

## Data Availability

The datasets used and/or analysed during the current study are available from the corresponding author on reasonable request.

## Abbreviations

CAD: Coronary artery disease
CI: Confidence interval
CKD: Chronic kidney disease
DAPT: Dual antiplatelet therapy
HCY: Homocysteine
HDL: High-density-lipoprotein
HR: Hazard ratio
ISR: In-stent restenosis
LAD: Left Anterior Descending artery
LDL: Low-density-lipoprotein
PAR%: Population attributable risk percentage
PAR: Population attributable risk
PCI: Percutaneous coronary intervention
TC: Total cholesterol
TG: Triglyceride
TPPA: *Treponema pallidum* Particle agglutination
TRUST: Toluidine red unheated serum test
